# Social network analysis for medical narcotics in South Korea: focusing on patients and healthcare organizations

**DOI:** 10.1186/s12913-024-11005-z

**Published:** 2024-05-07

**Authors:** Sang-Yoon Kim, Nam-Wook Cho

**Affiliations:** 1 Korea Institute of Drug Safety & Risk Management, 5 Fl., 30, Burim-Ro 169Beon-Gil, Dongan-Gu, Anyang-Si, Gyeonggi-Do Republic of Korea; 2 Department of Industrial Information Systems, Graduate School of Public Policy and IT, Seoul National University of Science & Technology, 232 Gongneung-Ro, Nowon-Gu, Seoul, 139-743 Republic of Korea; 3 Department of Industrial Engineering, Seoul National University of Science and Technology, 232 Gongneung-ro, Nowon-gu, Seoul, 139-743 Republic of Korea

**Keywords:** Drug safety, Medical narcotics, Narcotics Information Management System, Controlled substance, Social network analysis

## Abstract

**Background:**

Medical narcotics must be administered under medical supervision because of their potential for misuse and abuse, leading to more dangerous and addictive substances. The control of medical narcotics requires close monitoring to ensure that they remain safe and effective. This study proposes a methodology that can effectively identify the overprescription of medical narcotics in hospitals and patients.

**Methods:**

Social network analysis (SNA) was applied to prescription networks for medical narcotics. Prescription data were obtained from the Narcotics Information Management System in South Korea, which contains all data on narcotic usage nationwide. Two-mode networks comprising hospitals and patients were constructed based on prescription data from 2019 to 2021 for the three most significant narcotics: appetite suppressants, zolpidem, and propofol. Two-mode networks were then converted into one-mode networks for hospitals. Network structures and characteristics were analyzed to identify hospitals suspected of overprescribing.

**Results:**

The SNA identified hospitals that overprescribed medical narcotics. Patients suspected of experiencing narcotic addiction seek treatment in such hospitals. The structure of the network was different for the three narcotics. While appetite suppressants and propofol networks had a more centralized structure, zolpidem networks showed a less centralized but more fragmented structure. During the analysis, two types of hospitals caught our attention: one with a high degree, meaning that potential abusers have frequently visited the hospital, and the other with a high weighted degree, meaning that the hospital may overprescribe. For appetite suppressants, these two types of hospitals matched 84.6%, compared with 30.0% for propofol. In all three narcotics, clinics accounted for the largest share of the network. Patients using appetite suppressants were most likely to visit multiple locations, whereas those using zolpidem and propofol tended to form communities around their neighborhoods.

**Conclusions:**

The significance of this study lies in its analysis of nationwide narcotic use reports and the differences observed across different types of narcotics. The social network structure between hospitals and patients varies depending on the composition of the medical narcotics. Therefore, these characteristics should be considered when controlling medication with narcotics. The results of this study provide guidelines for controlling narcotic use in other countries.

## Background

Medical narcotics are essential drugs used for anesthesia, pain relief, sedation, and other therapeutic purposes in surgery and disease treatment. However, they also impose risks of misuse and abuse, as well as the potential to lead to more dangerous and addictive substances.

Therefore, many countries operate information systems that control and monitor medical narcotics to ensure their safe management. In South Korea, the Narcotics Information Management System (NIMS) implementation process commenced in May 2018 and was completed in July 2019. The NIMS ensures that all narcotic handlers digitally report all information on handling medical narcotics. Consequently, per patient, the average prescription of medical narcotics, including propofol, zolpidem, and appetite suppressants, decreased by 9.2% in 2019 [1].

Regulating hospitals that overprescribe narcotics is essential to prevent their misuse, as narcotic addicts have difficulty controlling themselves, which can lead to the use of more dangerous and addictive substances. Therefore, it is important to regulate medical institutions to prevent excessive prescriptions. However, using prescription counts alone to identify overprescribing hospitals has limitations as they may include medical institutions that legitimately use medical narcotics, resulting in a waste of administrative resources and potentially undermining proactive medical practices.

This paper proposes the application of social network analysis (SNA) to identify and prevent the excessive prescription of medical narcotics in both hospitals and patients, as well as address the issue of their misuse and abuse. To accomplish this objective, nationwide prescription data for three significant narcotics in South Korea were analyzed over two years, reported as NIMS, from July 2019 to June 2021. The timeframe was based on the full implementation of the NIMS Reporting Law in July 2019. In this study, we analyzed the three most significant narcotics, including appetite suppressants,^1^ zolpidem, and propofol,^2^ designated by the Standards for Safe Use of Medical Narcotics (SSUN) established by the Ministry of Food and Drug Safety(MFDS) in South Korea. According to the SSUN, appetite suppressants should be used for the purpose of obesity treatment for less than four weeks with a maximum of three months. zolpidem should be used at a maximum dose of 10mg per day for the treatment of insomnia for a duration not exceeding four weeks. Propofol is used for the purposes of general anesthesia and sedation, and it is desirable not to exceed its use more than once a month [2]. Although regulation is not mandatory, excessive prescription of these narcotics may result in the oversight of MFDS management. By analyzing national narcotic use reports and the differences observed in different types of narcotics, this study provides a methodology to effectively identify hospitals that overprescribe medical narcotics.

### Healthcare information system

Synthetic opioid analgesics are exhibiting a rising incidence of abuse and addiction worldwide [3]. Consequently, numerous nations have implemented an information system to manage narcotic use and control substances.

Fifty states in the United States operate individual Prescription Drug Monitoring Programs (PDMP) per the Model Prescription Monitoring Program. In Canada, seven provinces operate computing systems to monitor narcotic prescriptions under the Narcotics Safety and Awareness Act. In Australia, Tasmania operates a Drug and Poison Information System Online Remote Access (DORA) to manage prescriptions for narcotics and poisonous substances [4–6]. The MFDS and the Korea Institute of Drug Safety and Risk Management (KIDS) implemented a narcotic-handling reporting system through the NIMS in May 2018 to manage narcotics safely in Korea. The NIMS requires pharmaceutical companies, wholesalers, hospitals, and pharmacies handling narcotics to report all processes, including import, production, sales, purchasing, dispensing, and disposal, through computerized reporting according to Article 11 (Reporting of Handling Narcotic Drugs) of the Narcotics Control Act [1].

### Narcotics analysis by SNA

Analyzing prescription patterns between hospitals and patients is a relational behavior; therefore, several studies have proposed using SNA [7–9]. In social science, the structural approach, which is based on the analysis of interactions between social actors, is called SNA, which is grounded in the intuitive notion that the patterning of social ties in which actors are embedded has significant consequences for those actors [10].

SNA has proven useful in analyzing the relationship between lifestyle and drug misuse [11]. SNA was useful in investigating and analyzing the correlation between drug misuse, child abuse, mental illness, and other behaviors. Paul et al. [12] examined the blackbird crime network associated with cannabis cultivation in the Netherlands. They stated that criminal networks are not as well known as other types of networks and that with the increase in data availability, scientific evidence can be provided on how criminal networks operate and respond and that they can be blocked. They confirm the importance of brokers and discover that the network operates around a few central actors. Rémi [13] used SNA to analyze price differences in narcotic trafficking between countries, stating that narcotics trafficking is an illegal activity involving producers, distributors, and banned products. Carl et al. [14] used SNA to examine the social relationships of 293 individuals who used narcotics in Baltimore, Maryland, and evaluated the relationship between network characteristics and HIV risk behavior. Malcolm [15] claimed that centrality in network analysis helps identify, remove, or monitor key players in neutralizing crime, and equivalence refers to the ability to replace or interact with nodes that are precisely connected in the same way. However, existing studies of SNA on narcotics have observed and analyzed data collected through interviews targeting specific cities or states. Expanding the scope of SNA analysis to the national level provides a more comprehensive understanding of the phenomenon, facilitating informed decision-making and policy formulation.

## Methods

### Data source

The data used in this study are narcotics prescriptions reported to NIMS from July 2019 to June 2021, and we extracted data exceeding SSUN for the analysis. Tables 1 and 2 summarize all prescriptions of medical narcotics used in this study [16]. The data indicated that, on average, one in 2.8 Koreans got a prescription for medical narcotics each year. It should be noted that this statistic includes medically appropriate narcotic use.^3^

**Table 1:** Prescriptions for medical narcotics in South Korea during 2019 ~ 2021

Classification	2019	2020	2021
Number of patients	18,502,227	17,475,493	18,844,312
Number of prescriptions	99,677,125	99,939,580	103,380,489
Average number of prescriptions per patient (Number of prescriptions/Number of patients)	5.39	5.72	5.49

**Table 2:** Prescriptions for appetite suppressants, zolpidem, and propofol in South Korea during 2019–2021

Classification	Appetite suppressants	Zolpidem	Propofol
Number of patients	1,294,933	1,817,204	8,843,562
Number of prescriptions	6,109,202	11,736,547	10,667,079
Average number of prescriptions per patient (Number of prescriptions/Number of patients)	4.72	6.46	1.21

An analysis of the overall usage of the three narcotics reported through the NIMS in Korea is presented in Table 2 [16].

**Fig. 1:**
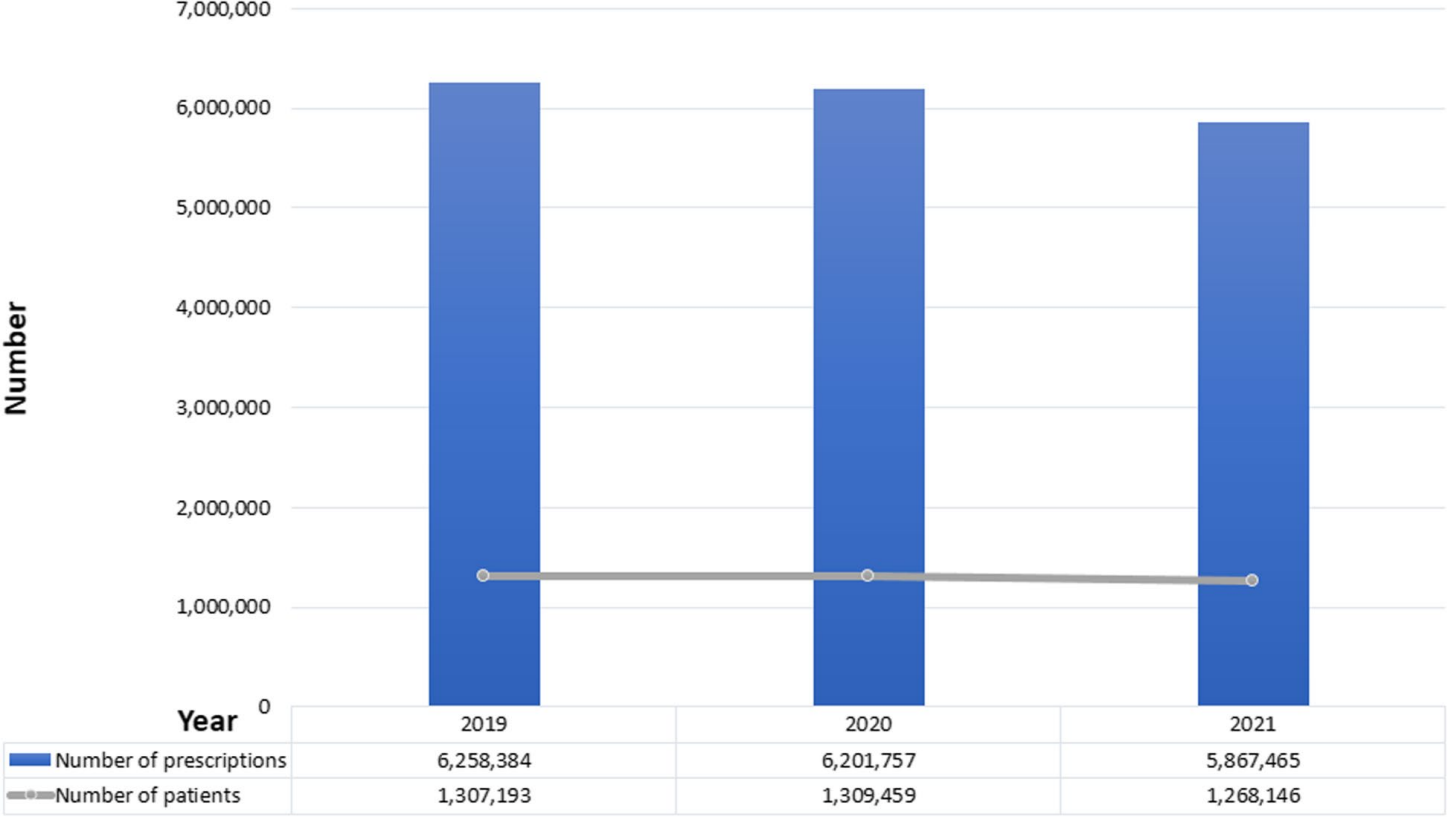
Number of patients and prescriptions for appetite suppressants

**Fig. 2:**
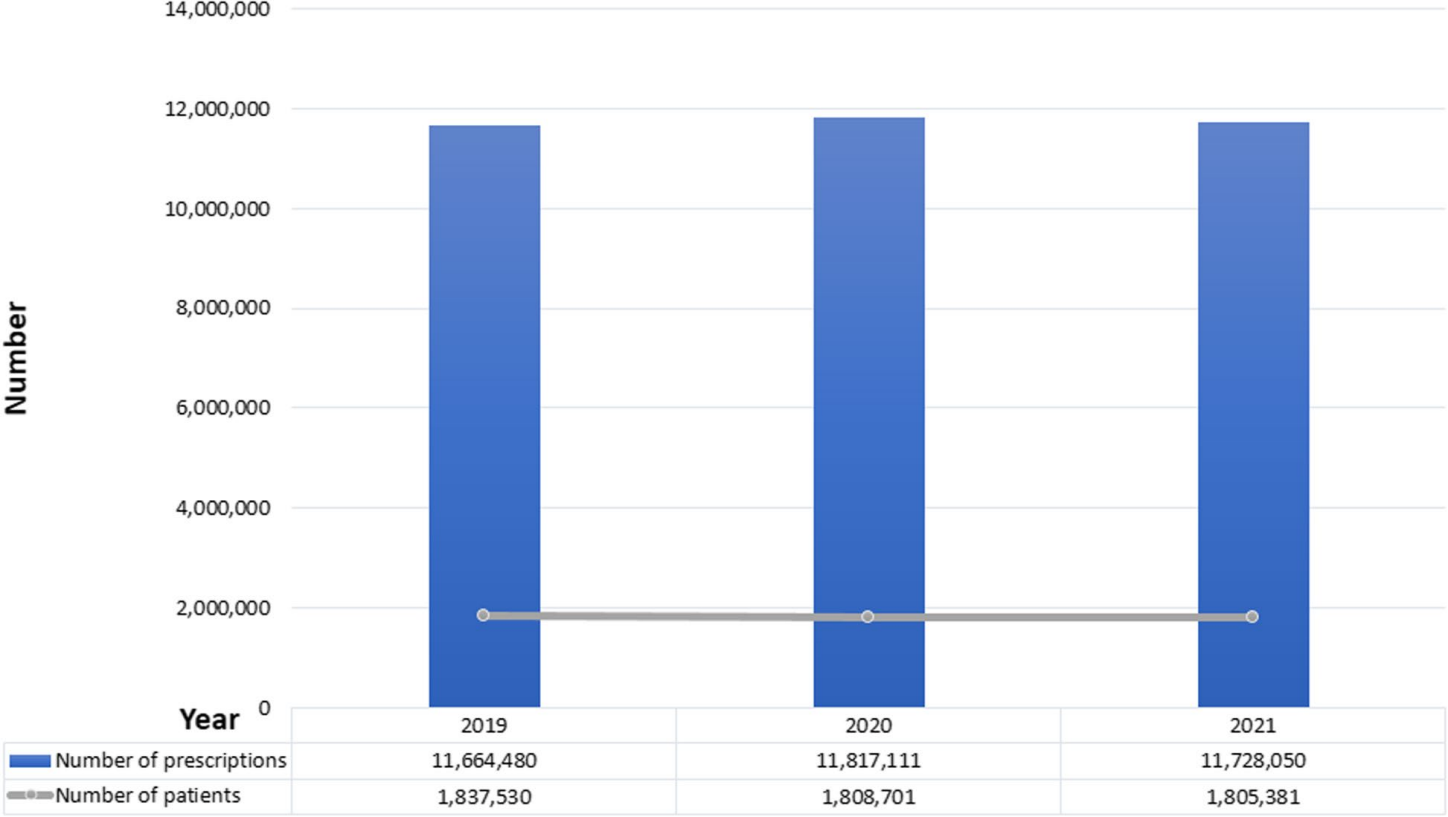
Number of patients and prescriptions for zolpidem

**Fig. 3:**
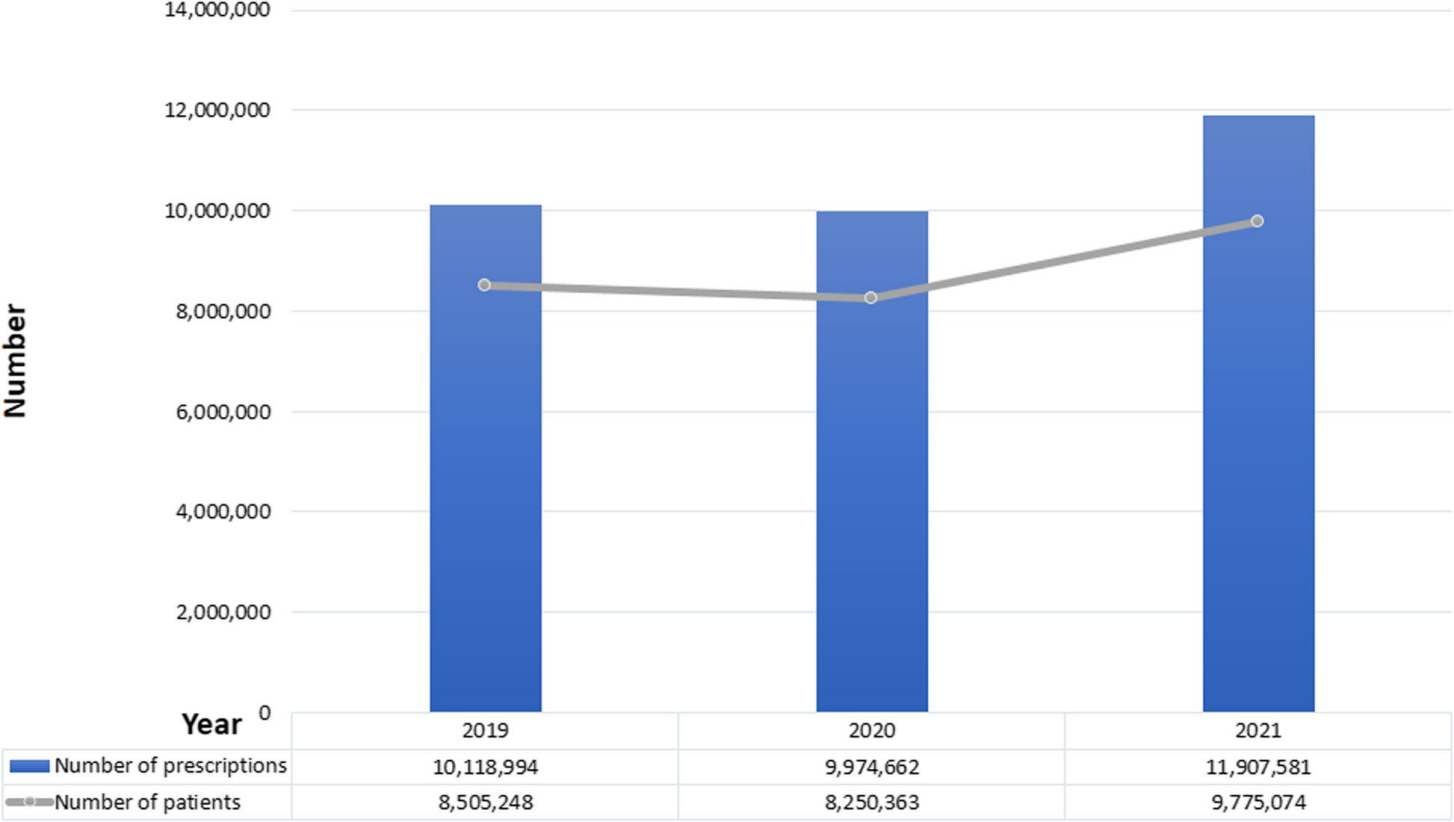
Number of patients and prescriptions for propofol

As shown in Table 2, the average number of prescriptions per patient is 6.45 for zolpidem, followed by 4.71 for appetite suppressants, and 1.2 for propofol, suggesting that propofol prescription is relatively under control. Figure 1 shows the prescriptions for appetite suppressants. The number of patients and prescriptions for appetite suppressants decreased around the time of the COVID-19 pandemic. Figure 2 shows the prescriptions issued for zolpidem. There was a slight increase in prescriptions in 2020; however, the overall trend was downward. However, propofol, as shown in Fig. 3, has shown an increase in terms of patient use and prescriptions around the time of the pandemic, with a significant increase of 19% in prescriptions and 18% in patients in 2021 compared to the previous year.

## Research design

This study aimed to identify and prevent the overprescription of medical narcotics in hospitals and patients and to mitigate their misuse and abuse. Thus, we first extracted the dosage data that exceeded the medical narcotic safety standards of the SSUN. However, exceeding medical narcotic safety standards does not necessarily mean overprescription or addiction. It should be noted that these drugs can be prescribed for medical purposes at the doctor’s discretion. Social networks based on prescriptions were constructed to further analyze the data based on medical narcotics.

First, undirected two-mode networks, also known as bipartite or bimodal networks, comprising hospitals and patients, were investigated. As shown in Table 3, data have been preprocessed to model the two-mode network. The node list consists of hospitals and patients, and the edges represent prescription relationships between hospitals and patients. During this process, data was anonymized in compliance with relevant laws in South Korea.

**Table 3:** Example of data structure

Classification	Nodes	Lines (Edges)
Item	ID, Category	Source, Target, Type
Example	H-01, Hospital H-02, Hospital P-01, Patient P-02, Patient …	P-01, H-01, undirected P-01, H-02, undirected P-02, H-01, undirected …

Second, patient nodes were removed from the two-mode network using matrix multiplication, transforming it into a one-mode network consisting of hospital-to-hospital connections, also known as a monopartite network. The two-mode network helps uncover social relationships among hospitals that were previously unidentified [17]. SNA analysis was performed using versions Gephi 0.9.7 and Ucinet 6.758 versions. To interpret the results of the SNA analysis, we conducted interviews with five pharmacists with experience working in pharmaceutical companies, hospitals, and pharmacies.

## Results

### Two-mode network analysis

Table 4 presents the current status of narcotic prescriptions that exceed the SSUN for medical narcotics. Interestingly, the standard deviation of the prescription count for propofol was 27.2, with a mean prescription count of 7.9, indicating a skewed distribution.

**Table 4:** The number of prescriptions per patient exceeding the SSUN

Classification	Appetite suppressants	Zolpidem	Propofol
Average	5.6	3.8	7.9
Standard deviation	6.3	8.3	27.2

**Fig. 4:**
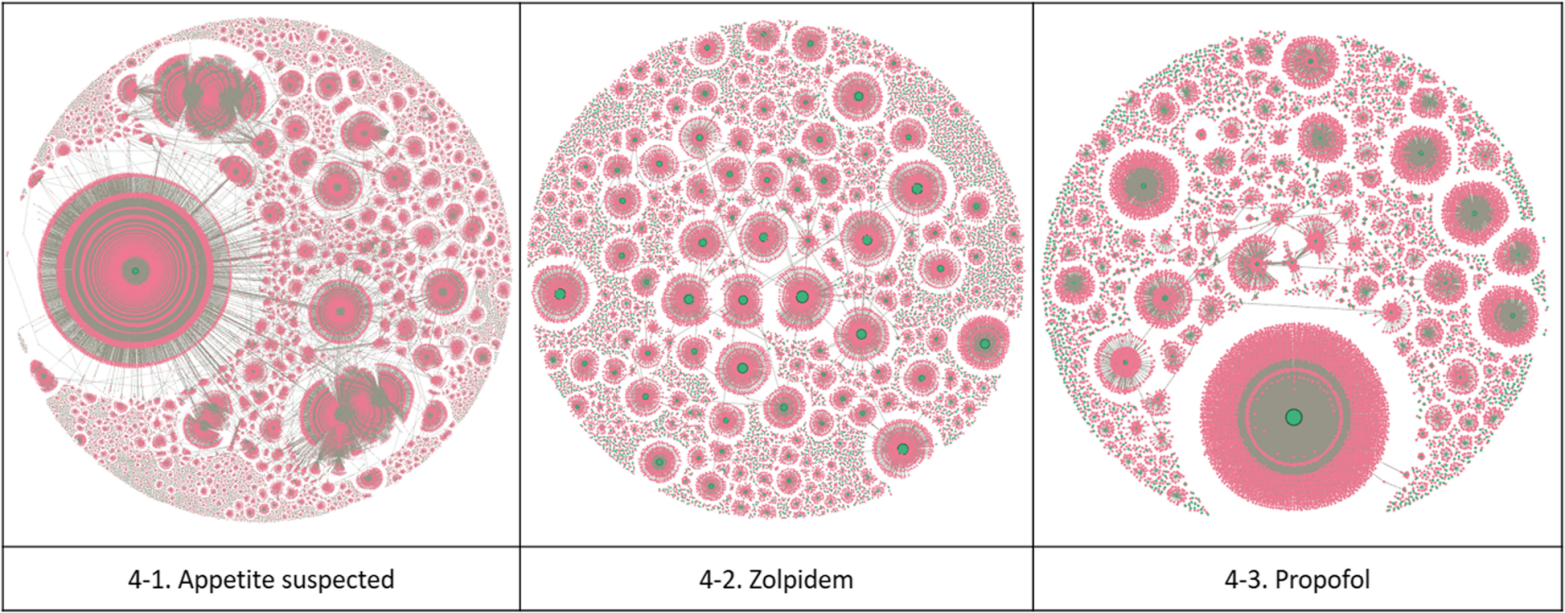
Two-mode network(hospital—Patient—hospital)

Figure 4 illustrates the two-mode networks of three narcotics: Fig. 4–1 depicts the network for appetite suppressants, Fig. 4–2 for zolpidem, and Fig. 4–3 for propofol, respectively. Note that hospital nodes are colored green, while patient nodes are colored pink. Appetite suppressants and propofol showed the characteristic of connecting large communities to one giant community. In contrast, the zolpidem network was more fragmented, with multiple communities of similar sizes.

Table 5 presents the network characteristics of the two-mode network analysis. The average degree is defined as the average number of connections. Although appetite suppressants had the highest value (2.032), no significant difference was found among the narcotics. The average weighted degree refers to the strength of the connections between the connected nodes, as measured by the number of prescriptions issued. Propofol had the highest average weighted degree at 14.712.

**Table 5:** Results of two-mode network analysis

Network characteristics	Appetite suppressants	Zolpidem	Propofol
Average degree	2.032	1.812	1.875
Average weighted degree	10.523	6.714	14.712
Network diameter	23	26	16
Modularity	0.857	0.981	0.967
Modularity-Number of communities	4631	2695	951
Average path length	13.971	10.859	3.261

The network diameter is defined as the longest length of the shortest path between pairs of network nodes. Among the three narcotics, zolpidem had the longest diameter. Modularity, which is also known as groups, clusters, or communities, is a method for measuring the structure of communities in large networks. A higher value indicated that the connections between nodes within a module were denser, whereas those between nodes in different modules were sparse.

Among the three narcotics, the number of communities was highest for appetite suppressants, but modularity was highest for zolpidem, indicating that zolpidem had the largest count of small-to-medium-sized communities, as illustrated in Fig. 4.

The average path length is defined as the average path distance between all the pairs of nodes. The appetite suppressant had the longest average path length of 13.971, whereas propofol had the shortest path length of 3.261.

Prior to the detailed analysis, let us explain the metrics used in our study. *Degree centrality*, referred to as *degree* hereafter, is defined as the number of connections that a node has to other nodes. On the other hand, *weighted degree centrality*, referred to as *weighted degree* hereafter, considers the strength of the connections between nodes. Thus, the degree of a hospital refers to the number of patients who visit the hospital, and the weighted degree of a hospital refers to the number of prescriptions. For example, if a hospital has a high-weighted degree but a low degree, this may indicate overprescription. Betweenness centrality refers to a node that is likely to play the role of a broker because it lies in the middle of the path between nodes. Eigenvector centrality refers to the connection of a node to important nodes.

### Appetite suppressant

The two-mode network of appetite suppressants comprises 98,140 nodes and 99,731 edges. The two-mode network of appetite suppressants in Fig. 4–1 shows a large community centered around a specific hospital; overall, it exhibits the characteristic of connecting medium-sized communities to large communities. Modularity is the lowest among narcotics due to its strong tendency to converge in certain hospitals. The average degree of all three narcotics was not significantly different, ranging from 1.8 to 2.0. The average weighted degree of appetite suppressants was 10.523, the second highest, implying a high tendency for patients concerned about the misuse of appetite suppressants to receive prescriptions from a few specific hospitals rather than multiple hospitals.

One of the most important objectives of this study is to understand the differences between hospitals where many patients gather and those that issue many prescriptions to specific patients. Table 6 presents the top ten hospitals based on the network centralities of the appetite-suppressant network. A significant overlap was observed between hospitals frequently visited by potential abusers and those with excessive prescriptions. Of the hospitals ranked in the top 1% based on degree and weighted degree, 84.6% were identical.

**Table 6:** Appetite-suppressant network analysis (top 10 hospitals)

Degree		Weighted Degree		Betweenness Centrality		Eigenvector Centrality	
Hospital	Score	Hospital	Score	Hospital	Score	Hospital	Score
H-4875	26,106	H-4875	184,792	H-4875	2,255,086,459	H-4875	1.0000
H-25895	3980	H-25895	21,232	H-5663	475,157,120	H-25895	0.1349
H-5663	3898	H-5663	18,707	H-25895	404,884,767	H-5663	0.1324
H-22913	2951	H-19210	16,758	H-19210	383,559,980	H-22913	0.1004
H-19210	2899	H-22542	12,849	H-22542	175,859,800	H-19210	0.0967
H-22542	2772	H-22913	10,822	H-2651	165,097,030	H-22542	0.0940
H-11751	1649	H-11751	9310	H-22913	146,228,031	H-11751	0.0546
H-8814	1527	H-2651	7967	H-8814	140,276,105	H-8814	0.0505
H-2651	1194	H-8814	6092	H-11751	137,473,147	H-2651	0.0394
H-21273	1095	H-6931	5481	H-6931	119,739,755	H-21273	0.0362

### Zolpidem

The zolpidem network consists of 26,536 nodes and 24,036 edges. Figure 4–2 shows the zolpidem two-mode network, which has a different community structure than that of appetite suppressants. Although the average degrees and average weighted degrees were the lowest, the network diameter was the highest, indicating that the nodes were most distantly connected. The total prescription count was the highest, but the number of prescriptions exceeding the SSUN was the lowest. Because the average degree and average weighted degree were the lowest, it appeared to be the best managed among the three narcotics.

Table 7 presents the top ten hospitals based on the network statistics of the zolpidem SNA. Of the hospitals ranked in the top 1% based on degree and weighted degree, 82.1% were identical, which indicates a notable intersection between hospitals experiencing a high volume of patient visits and those that engage in overprescribing practices, similar to appetite suppressants.

**Table 7:** Zolpidem network analysis (Top 10 hospitals)

Degree		Weighted Degree		Betweenness Centrality		Eigenvector Centrality	
Hospital	Score	Hospital	Score	Hospital	Score	Hospital	Score
H-8241	650	H-17856	6639	H-30462	34,219,084	H-8241	1.0000
H-10239	575	H-8241	3085	H-8241	28,668,821	H-10239	0.7882
H-31311	566	H-17605	3030	H-16800	28,121,280	H-31311	0.7652
H-30462	543	H-23237	2702	H-9267	26,054,357	H-30462	0.7111
H-23237	507	H-27032	2525	H-17857	24,140,286	H-23237	0.6265
H-15821	501	H-10239	2429	H-17605	11,952,374	H-15821	0.6098
H-16800	490	H-16800	2146	H-23237	9,989,702	H-16800	0.5911
H-27170	468	H-30462	2141	H-31311	7,585,979	H-27170	0.5396
H-4084	450	H-31311	2090	H-23024	7,531,404	H-4084	0.5044
H-30459	444	H-15821	1980	H-25232	6,559,112	H-30459	0.4928

### Propofol

Owing to its rapid onset of action and short duration, propofol can be used as an intravenous sedative and hypnotic drug. As an easily obtainable and misused drug, it has significant potential. Consequently, Korea became the world's first country to designate it as a controlled substance. In February 2011, the MFDS designated propofol as a psychotropic drug, making it mandatory to regulate its use [18].

The propofol network consisted of 14,368 nodes and 13,467 edges. As seen in the two-mode network of propofol in Fig. 4–3, a few large communities were observed. The average weighted degree was highest at 14.712.

Table 8 presents the top 10 hospitals based on the network statistics of propofol SNA. Of the hospitals ranked in the top 1% based on the degree and weighted degree, only 30.0% were identical. This shows the differences between hospitals with high visits and those overprescribing, unlike appetite suppressants and zolpidem. Consequently, it suggests that propofol should be managed differently from other narcotics.

**Table 8:** Propofol network analysis (Top 10 hospitals)

Degree		Weighted Degree		Betweenness Centrality		Eigenvector Centrality	
Hospital	Score	Hospital	Score	Hospital	Score	Hospital	Score
H-2089	3329	H-4	8012	H-2089	5,592,764	H-2089	1.0000
H-2101	437	H-6729	7117	H-3302	1,196,491	H-2101	0.0747
H-6126	378	H-29	6887	H-4	917,869	H-6126	0.0638
H-8041	333	H-4624	6250	H-37	810,328	H-8041	0.0556
H-14	324	H-14	5040	H-17	625,076	H-14	0.0540
H-493	317	H-3280	5015	H-14	615,477	H-493	0.0527
H-37	305	H-17	4235	H-3291	611,817	H-37	0.0506
H-29	269	H-2089	3579	H-29	572,070	H-29	0.0442
H-5929	265	H-5666	3295	H-6729	230,520	H-5929	0.0435
H-1758	220	H-6708	3051	H-3278	170,561	H-1758	0.0358

### One-mode network analysis

The two-mode network analysis revealed that networks of appetite suppressants and propofol were concentrated in specific hospitals, whereas zolpidem networks were more fragmented. For a more in-depth analysis of hospital networks, this section presents an analysis of the one-mode networks of hospitals converted from the two-mode networks in the previous section.

### Appetite suppressant

**Fig. 5:**
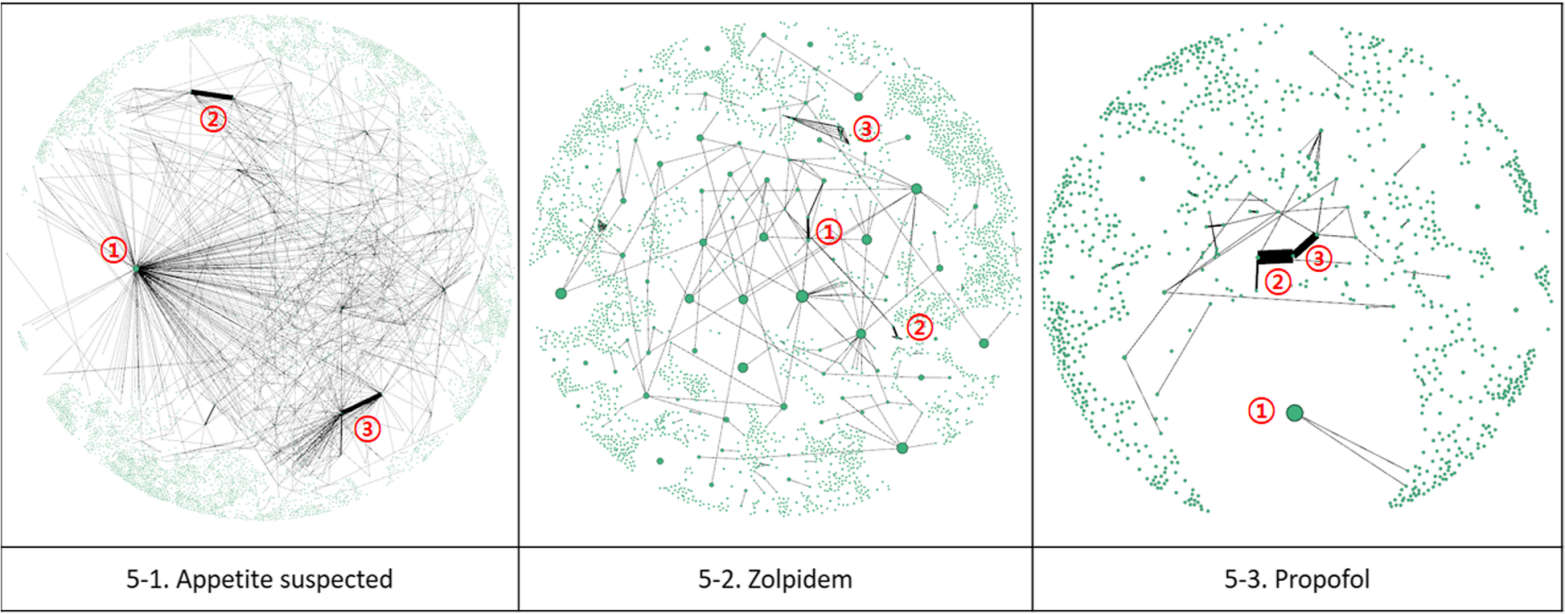
One-mode network(hospital—hospital)

The one-mode network for appetite suppressants is shown in Fig. 5–1. The symbol ① denotes a hospital that constitutes the largest community within the network. Nevertheless, it is discernible that the edges corresponding to ② and ③ are thicker, indicative of more frequent prescription occurrences for specific patients. The one-mode network discerns hospitals with frequent patient visits (①) and those where prescriptions are commonly dispensed to patients (②, ③).

**Fig. 6:**
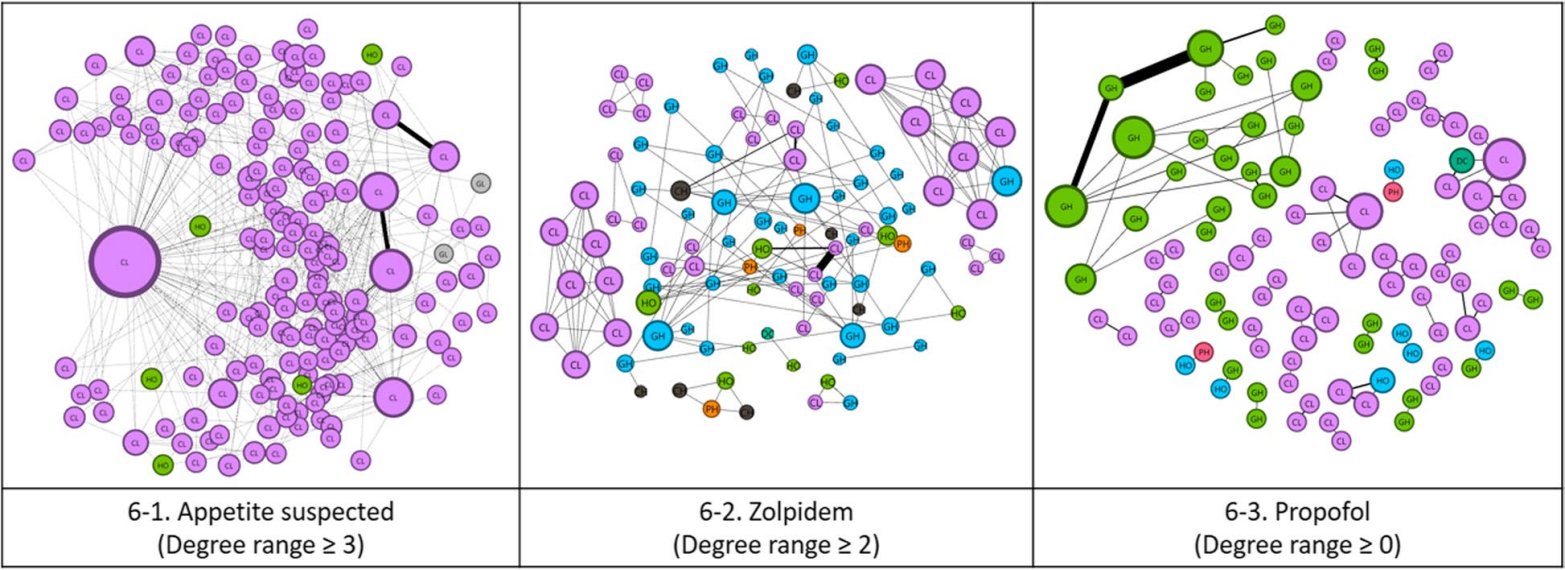
One-mode network by hospital size. (GH: General hospital, PH: Psychiatric hospital, OMH: Oriental medicine hospital, CH: Convalescent hospital, DC: Dental clinic, PHCE: Public health center, PHCL: Public health clinic, PHU: Public health units, HO: Hospital, CL: Clinic)

**Fig. 7:**
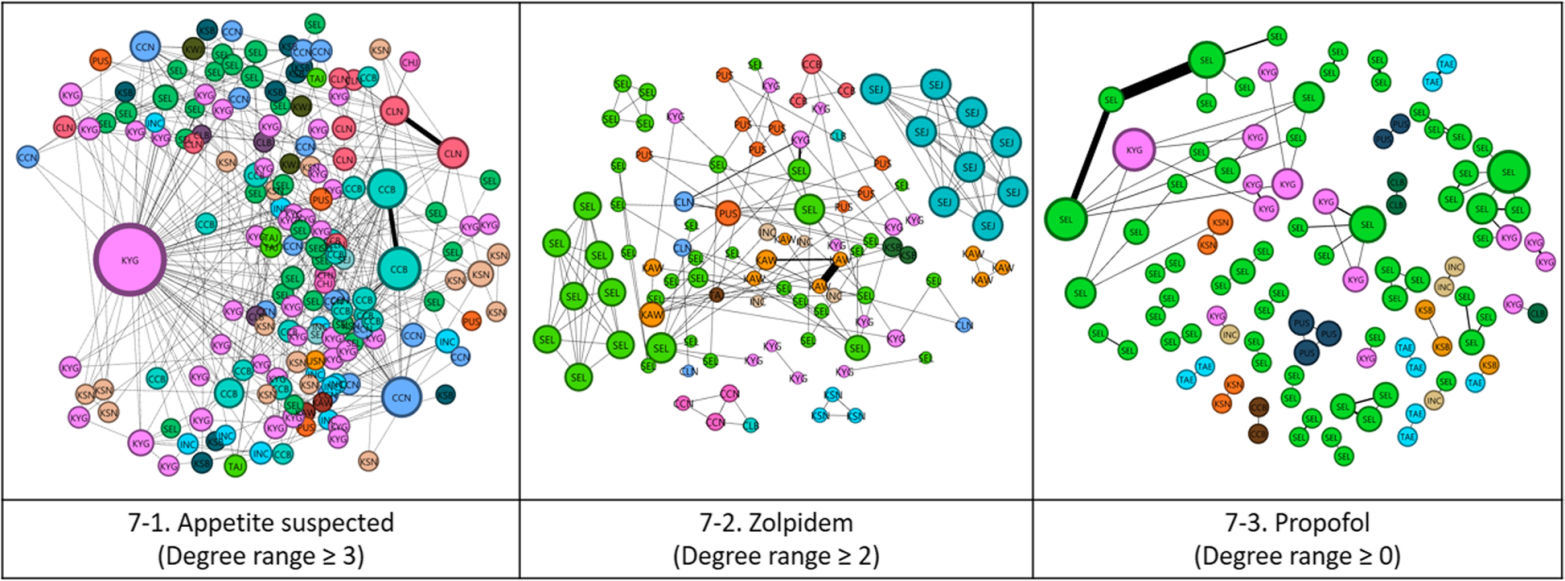
One-mode network by region. (SEL: SEOUL, PUS: PUSAN, TAE: TAEGU, INC: INCHON, KWJ: KWANGJU, TAJ: TAEJON, USN: ULSAN, KYG: KYONGGI, KAW: KANGWON, CCB: CHUNGBUK, CCN: CHUNGNAM, CLB: CHONBUK, CLN: CHONNAM, KSB: KYONGBUK, KSN: KYONGNAM, CHJ: CHEJU)

Figure 6–1 presents the appetite suppressant network according to hospital size, which shows that clinics have the largest share of the network and the highest weighted degree. Figure 7–1 presents the appetite suppressant network by region. It has been observed that patients using appetite suppressants were the most likely to visit multiple locations and, therefore, were at a higher risk of doctor shopping than other medical narcotics.

### Zolpidem

Figure 5–2 illustrates the one-mode network of zolpidem. In this figure, symbols ①, ②, and ③ exhibit thicker edges compared to other connections. This enabled us to establish priority for hospitals to be inspected first in networks of similar size.

Figure 6–2 presents a zolpidem network based on hospital size. It has been observed that clinics had the largest share of the network and the highest weighted degree, followed by general hospitals. Figure 7–2 presents the zolpidem network by region and shows that communities were more likely to form within similar regions.

### Propofol

The one-mode network of propofol is depicted in Fig. 5–3. Symbol ① constitutes a significant community within the two-mode network, whereas symbols ② and ③ frequently prescribe propofol to specific patients.

Figure 6–3 presents a propofol network based on hospital size. Clinics comprised the largest share of the network, followed by general hospitals, which had the second largest share. Unlike zolpidem, general hospitals had the highest weighted degrees. Figure 7–3 presents the propofol network by region. Similar to the case of zolpidem, communities were more likely to form within similar regions.

## Discussion

In this study, we analyzed hospital and patient social networks for three types of medical narcotics—appetite suppressants, zolpidem, and propofol—that exceeded the SSUN limit and were used in Korea for two years. First, in the two-mode network analysis results (Refer to Fig. 4), patients who received many prescriptions tended to cluster around hospitals that could easily prescribe narcotics. The appetite suppressants had an average of 2.032 connected nodes, with an average of 10.523 prescriptions issued per node. Zolpidem had 1.812 connected nodes, with an average of 6.714 prescriptions issued per node, whereas propofol had 1.875 connected nodes, with an average of 14.712 prescriptions issued per node. Second, clustering around specific hospitals was related to specific medical narcotics. Specifically, appetite suppressants and propofol showed high clustering tendencies, whereas zolpidem showed relatively low clustering tendencies. Finally, SNA identified two types of hospitals requiring attention: One is frequently visited by patients with a suspected overdose, and the other is suspected to be overprescribed. In addition, although clinics had the largest share of the network and the highest weighted degree of appetite suppressants, communities with similar hospital sizes were observed for zolpidem and propofol. Networks of appetite suppressants were geographically dispersed, implying a higher risk of doctor shopping than for other medical narcotics.

One of the main contributions of this study is that it reveals the different social network structures of medical narcotics requiring different approaches to control them on a national scale. In particular, based on the top 1% criteria of degree and weighted degree, appetite suppressants and zolpidem presented a high percentage of matching hospitals (84.6% and 82.1%, respectively), whereas propofol presented only a 30.0% match, indicating that it needs to be managed differently from other narcotics. The prescription patterns of medical narcotics are shown in Table 9.

**Table 9:** Prescription patterns of medical narcotics

	Low degree	High degree
Low weighted degree	-	appetite suppressants
High weighted degree	propofol	appetite suppressants, zolpidem, propofol

To better understand the reasons for the prescription patterns of medical narcotics, we conducted interviews with five pharmacists with experience working in pharmaceutical companies, hospitals, and pharmacies. A summary of the interview is presented below.

Zolpidem is likely to be used for calming, sleep, and other treatment purposes, and care should be taken to prevent the misuse of appetite suppressants and propofol. Appetite suppressants are likely to be easily prescribed in hospitals for cosmetic purposes and other reasons. Propofol appears to be used for health checkups in hospitals where many patients are seen, and it has been suggested that hospitals that prescribe a large amount of propofol to specific patients should be checked.

However, exceeding the SSUN for medical narcotics does not necessarily mean that the prescription is excessive or that the patient is addicted. Medical professionals may prescribe narcotics for medical purposes based on their professional judgment.

We propose the following measures to reflect the social network characteristics of narcotics and identify hospitals for inspection.

$$Narcotics\;Safety\;Index=\frac{Weighted\;degree}{Degree}$$

The Narcotics Safety Index can be used to prioritize hospitals for inspection. The MFDS analyzed data from the NIMS to send doctors a report titled "Guidance for the Safe Use of Medical Narcotics." As a result of the report, it was observed that the average prescription quantity per patient decreased by 9.2% [19]. Despite these achievements, merely managing based on the number of patient visits to hospitals or the number of prescriptions can lead to unintended consequences such as undermining proactive medical practices or wasting administrative resources [20]. Therefore, utilizing scientific decision-making through SNA analysis and the Narcotics Safety Index can contribute to the efficient formulation and implementation of government policies.

In this study, we analyzed the social network structure of medically prescribed narcotics at the national level. Through SNA analysis, we discovered the prescription characteristics of medical narcotics according to their components. Additionally, we found that medical institutions' size and regional characteristics vary depending on the components of medical narcotics. Based on these findings, we proposed a Narcotics safety index. Despite the contributions of this study, it has limitations. This paper is based on the data from 2019 to 2021. Therefore, future studies should collect data from 2022 and beyond to conduct cohort and time series analyses.

## Conclusion

This study used SNA techniques to analyze hospital and patient characteristics that exceeded the SSUN set by the MFDS. By examining the social network differences in the use of appetite suppressants, zolpidem, and propofol over two years in South Korea, this study was able to identify hospitals where patients were suspected of medical shopping and hospitals that prescribed heavily to specific patients. Law enforcement agencies can utilize the analytical techniques and results obtained in this study for inspection, monitoring, and guiding narcotic use to prevent misuse and abuse. This study is expected to help protect public health and contribute to the development of a safer society by preventing narcotic misuse.

## Data Availability

The raw data used in this paper is not publicly available under Article 11–5 of the Korean Narcotics Control Act. Statistical data can be provided for research, investigation, and education on drug misuse upon reasonable request from the corresponding author.

## Abbreviations

KIDS: Korea Institute of Drug Safety and Risk Management
MFDS: Ministry of Food and Drug Safety in Korea
Narcotics: Medical narcotics
NIMS: Narcotics Information Management System
SNA: Social network analysis
SSUN: Standards for safe use of medical narcotics

## Footnotes

1 The appetite suppressants include five substances, namely Lorcaserin, Mazindol, Amfepramone, Phendimetrazine, and Phentermine.

2 Propofol is classified as an antipsychotic drug in South Korea.

3 Those who are enrolled in workplace health insurance in Korea are required to undergo regular upper or lower gastrointestinal endoscopy once every 1–2 years. Narcotics such as zolpidem and propofol may be used for sedation during the procedure.
